# Supplementary material for: Impact of tRNA-induced proline-to-serine mistranslation on the transcriptome of Drosophila melanogaster
Source: G3 (Bethesda). 2024 Jul 11;14(9):jkae151. doi: 10.1093/g3journal/jkae151 (PMC11373654; doi:10.1093/g3journal/jkae151)
Supplement: jkae151_Supplementary_Data [file jkae151_supplementary_data.zip › Supplemental_File_S2_G3-2024-405018.docx]

**Extended Methods**

Parameters and commands specified for the programs used as part of the RNA-sequencing pipeline are as follows:

*Trimmomatic v0.39*

Remove poor-quality bases and Illumina adapter sequences:
java -jar trimmomatic-0.39.jar PE input_forward.fq.gz input_reverse.fq.gz output_forward_paired.fq.gz output_forward_unpaired.fq.gz output_reverse_paired.fq.gz output_reverse_unpaired.fq.gz ILLUMINACLIP:TruSeq3-PE.fa:2:30:10:2:keepBothReads LEADING:3 TRAILING:3 MINLEN:36

*STAR v2.7.9a*

Create genome index:

STAR –-runThreadN 4 –-runMode genomeGenerate –-genomeDir ./DrosophilaGenome –-genomeFastaFiles ./dmel-all-chromosome-r6.41.fasta –-sjdbGTFfile ./dmel-all-r6.41.gtf –-sjdbOverhang 149

Align reads to genome:

STAR --runThreadN 4 --genomeDir ./DrosophilaGenome/Index --readFilesIn ./Trimmed/Reads_filtered_1P ./Trimmed/Reads_filtered_2P --outFileNamePrefix ./BAMfiles/Reads_paired --outSAMtype BAM Unsorted

STAR --runThreadN 4 --genomeDir ./DrosophilaGenome/Index --readFilesIn ./Trimmed/READS_filtered_1U --outFileNamePrefix ./BAMfiles/READS_oneU --outSAMtype BAM Unsorted

*FeatureCounts v2.0.0*

Count reads that map to each gene

featureCounts -T 4 -p -B -a

./DrosophilaGenome/dmel-all-r6.41.gtf -o ./RNAseq_output.txt

./BAMfiles/PairedAlignedReads.bam

./Bamiles/UnpairedAlignedReads.bam

The featureCounts output was then used for DEseq2 analysis in RStudio. Code used to perform that analysis can be found in Supplemental file S3.

**Table S1.** Concentration and absorbance ratios of RNA extracted from all three replicates of ten 1–3-day old virgin male and female tRNA^Ser^_UGA_ and tRNA^Ser^_UGG, G26A_ flies. The second value in the replicate column refers to the first or second round of RNA extractions performed on those samples (the extraction before DNase treatment or the extraction following DNase treatment).

| Sample | Replicate | Concentration (ng/µL) | A260/A280 | A260/A230 |
| --- | --- | --- | --- | --- |
| tRNA^Ser^_UGA_ -Male | 1-1 | 984 | 2.016 | 2.196 |
| tRNA^Ser^_UGA_ -Female | 1-1 | 1346 | 2.071 | 2.199 |
| tRNA^Ser^_UGG, G26A_ -Male | 1-1 | 986 | 2.029 | 2.241 |
| tRNA^Ser^_UGG, G26A_ -Female | 1-1 | 1604 | 2.062 | 2.197 |
| tRNA^Ser^_UGA_ -Male | 2-1 | 804 | 2.051 | 2.083 |
| tRNA^Ser^_UGA_ -Female | 2-1 | 1486 | 2.105 | 2.366 |
| tRNA^Ser^_UGG, G26A_ -Male | 2-1 | 536 | 2.015 | 2.015 |
| tRNA^Ser^_UGG, G26A_ -Female | 2-1 | 1664 | 2.096 | 2.514 |
| tRNA^Ser^_UGA_ -Male | 3-1 | 496 | 2.033 | 2.138 |
| tRNA^Ser^_UGA_ -Female | 3-1 | 1322 | 2.072 | 2.241 |
| tRNA^Ser^_UGG, G26A_ -Male | 3-1 | 204 | 2.04 | 1.925 |
| tRNA^Ser^_UGG, G26A_ -Female | 3-1 | 1360 | 2.092 | 2.297 |
| tRNA^Ser^_UGA_ -Male | 1-2 | 520 | 2.000 | 2.047 |
| tRNA^Ser^_UGA_ -Female | 1-2 | 516 | 2.000 | 2.263 |
| tRNA^Ser^_UGG, G26A_ -Male | 1-2 | 358 | 1.989 | 1.967 |
| tRNA^Ser^_UGG, G26A_ -Female | 1-2 | 662 | 2.018 | 2.489 |
| tRNA^Ser^_UGA_ -Male | 2-2 | 608 | 1.987 | 2.068 |
| tRNA^Ser^_UGA_ -Female | 2-2 | 1100 | 2.022 | 2.321 |
| tRNA^Ser^_UGG, G26A_ -Male | 2-2 | 348 | 2.000 | 1.596 |
| tRNA^Ser^_UGG, G26A_ -Female | 2-2 | 1144 | 2.021 | 2.444 |
| tRNA^Ser^_UGA_ -Male | 3-2 | 266 | 1.985 | 1.511 |
| tRNA^Ser^_UGA_ -Female | 3-2 | 972 | 2.042 | 1.869 |
| tRNA^Ser^_UGG, G26A_ -Male | 3-2 | 172 | 1.870 | 1.458 |
| tRNA^Ser^_UGG, G26A_ -Female | 3-2 | 998 | 2.037 | 2.189 |

**Table S2**. RT-qPCR primers used in this study

| Primer name | Sequence |
| --- | --- |
| CG12057_qPCR_F | CGCTCCTCCATCAAGACCAT |
| CG12057_qPCR_R | ACAAGCAACACTAGCGACGA |
| fiz_qPCR_F | ACCCGTCGAATCTGAGTTGC |
| fiz_qPCR_R | CCCGATCCTCCCAGCATTTT |
| CG4650_qPCR_F | CGGACTTCTGACGAATGGGA |
| CG4650_qPCR_R | CGCTGCAGTCAGAACTAATTTTTCA |
| Pif1A_qPCR_F | GCCAAGTCGAAGGATCCCAA |
| Pif1A_qPCR_R | GTCCAGGTCCTGCAGTGTTT |
| CG1503_qPCR_F | TTTCCACCCATCCAAGACCC |
| CG1503_qPCR_R | GCAAAGTTTCCGACACCGAG |
| CG11911_qPCR_F | GTTGAGTTCACAAACGCCCC |
| CG11911_qPCR_R | AATGTAGGCCGACACCTTGG |
| aTub84B_qPCR_F | TGTCGCGTGTGAAACACTTC |
| aTub84B_qPCR_R | GGATGGAGTTGTAGGGCTCG |


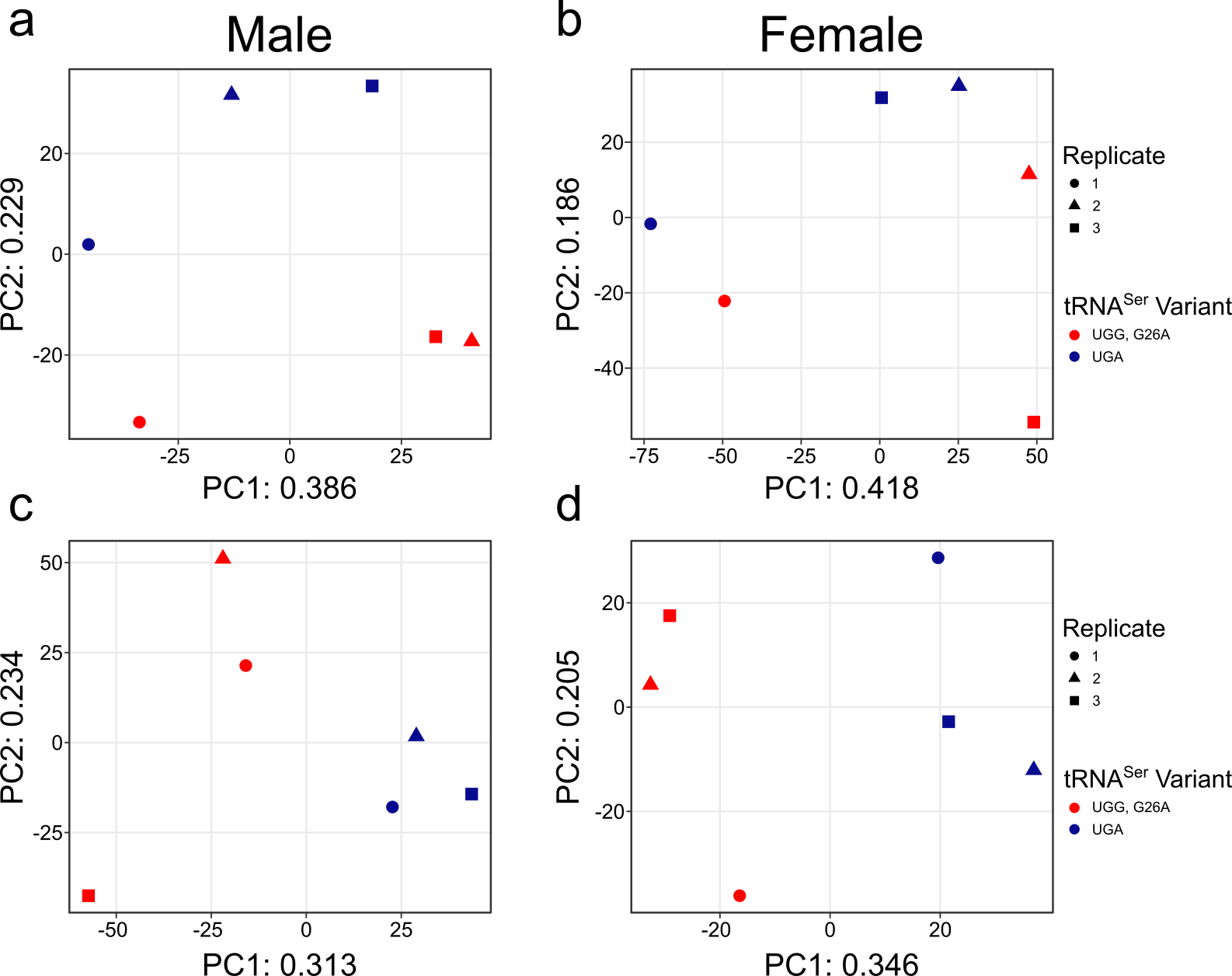


**Figure S1**. Principal component analysis (PCA) of all three replicates of tRNA^Ser^_UGA_ and tRNA^Ser^_UGG, G26A_ (P🡪S) centered log ratio transformed RNA sequencing data. **A)** PCA of male RNA sequencing data prior to batch correction. **B)** PCA of female RNA sequencing data prior to batch correction. **C)** and **D)** are the same as **A)** and **B)** but after batch correction using ComBat-seq. RNA from replicate 1 was extracted on a different day than RNA from replicates 2 and 3. Each point represents one replicate of 10 flies.

**
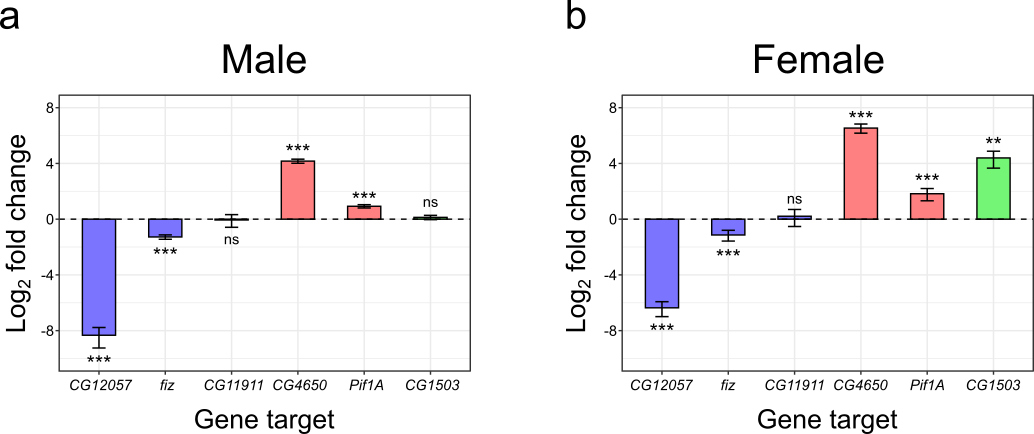
**

**Figure S2**. RT-qPCR quantification of expression changes for genes identified as differentially expressed from RNA-sequencing. Relative expression of the listed gene in **A)** male or **B)** female flies containing tRNA^Ser^_UGG, G26A_ (P🡪S) compared to flies containing tRNA^Ser^_UGA_­. Expression changes depicted in blue, red, and green were identified as downregulated, upregulated, or differentially regulated only one sex according to RNA-sequencing, respectively. Significance was calculated using an unpaired *t*-test in the Bio-Rad CFX Manager 3.0 software. Error bars represent the mean ± SEM. “ns” *P* ≥ 0.05, “**” *P* < 0.01, “***” *P* < 0.001.
